# A transgenic monkey model for the study of human brain evolution

**DOI:** 10.24272/j.issn.2095-8137.2019.031

**Published:** 2019-05-18

**Authors:** Lei Shi, Bing Su

**Affiliations:** 1 State Key Laboratory of Genetic Resources and Evolution, Kunming Institute of Zoology, Chinese Academy of Sciences, Kunming Yunnan 650223, China; 2 Primate Research Center, Kunming Institute of Zoology, Chinese Academy of Sciences, Kunming Yunnan 650223, China; 3 Center for Excellence in Animal Evolution and Genetics, Chinese Academy of Sciences, Kunming Yunnan 650223, China

Why humans have large brains with higher cognitive abilities is a question long asked by scientists. However, much remains unknown, especially the underlying genetic mechanisms. With the use of a transgenic monkey model, we showed that human-specific sequence changes of a key brain development gene (Primary microcephaly 1, *MCPH1*) could result in detectable molecular and cognitive changes resembling human neoteny, a notable characteristic developed during human evolution. This study was published in *National Science Review* ( Shi et al., 2019).

Neoteny has been hypothesized to help explain why we differ from our close relatives, such as the chimpanzee (*Pan troglodytes*) (Liu et al., 2012). Also called juvenilization, neoteny is the delay in or slowing down of physiological development of a species (Skulachev et al., 2017). Neoteny in humans is the retention of juvenile features into adulthood and is exaggerated compared to that in non-human primates. The neotenous human brain provides an extended window for the plasticity of the neural network, and therefore a longer time for learning, which may be crucial for the formation of human cognition (Bufill et al., 2011).

In the past two decades, comparative genomic studies have identified many candidate genes that carry human-specific sequence changes potentially contributing to human brain evolution (Bustamante et al., 2005; Clark et al., 2003). However, functional dissection of these candidate genes, particularly the phenotypic consequences of human-specific mutations, is lacking. As a model organism, transgenic mice (*Mus musculus*) have been used in the study of human brain function; for example, the study of *Foxp2* that carries two human-specific amino acid changes (Enard et al., 2009). However, compared to the human brain, the mouse brain is dramatically different in size, structure, developmental pattern, and function, and therefore is not an ideal model to study human brain evolution. In contrast, the rhesus monkey (*Macaca mulatta*), an Old-World primate species widely used in biomedical research, is a better animal model due to its high sequence similarity with humans (>93% for protein coding genes) (Yan et al., 2011; Zhang et al., 2014), yet relatively large phylogenetic distance (~25 million years of divergence from humans), which alleviates certain ethical concerns (Coors et al., 2010). These advantages have been demonstrated in the use of transgenic monkey models of human diseases (Chen et al., 2017; Liu et al., 2016; Luo et al., 2016; Qiu et al., 2019; Yang et al., 2008; Zhang et al., 2018).

*MCPH1* is a key gene regulating brain development in humans, and its truncate mutations can cause primary microcephaly (Jackson et al., 2002). Similarly, *MCPH1* knockout in mice and monkeys can recapitulate the microcephaly phenotype (Gruber et al., 2011; Ke et al., 2016), highlighting its critical role in mammalian brain development. In previous research, we demonstrated accelerated protein sequence changes in *MCPH1* during primate evolution, especially in the lineage leading to the origin of our own species (Wang & Su, 2004). Furthermore, *in vitro* functional analysis showed that human-specific *MCPH1* changes resulted in functional divergence between human and non-human primates (Shi et al., 2013). Such lines of evidence raise the question as to what phenotypic changes would appear following the introduction of human *MCPH1* copies into the rhesus monkey genome.

Using lentivirus delivery, we successfully generated 11 transgenic monkeys carrying 2–9 copies of human *MCPH1*. Brain image analysis showed that the transgenic monkeys had similar brain volume and cortex thickness as the controls, but their relative brain volume and gray matter percentage were higher. Importantly, the transgenic monkeys exhibited delayed cortex gray matter development compared to the controls. Furthermore, the transgenic monkeys had more immature and fewer mature neurons and glial cells than the controls, which, according to the transcriptome data, was likely caused by the suppression of many neuron maturation- and neuron differentiation-related genes. This observation was further illustrated by detailed analysis of developing brain laminas at the neurogenesis peak during fetal development.

Finally, to test whether the observed delay in brain development had any impact on behavior and cognition, we tested working memory in the transgenic monkeys using delayed matching-to-sample tasks. Notably, the transgenic monkeys exhibited better working memory and shorter reaction time than the controls, suggesting that neotenous brain development in the transgenic monkeys was beneficial for the formation of cognitive abilities, confirming the proposed evolutionary advantage of human neoteny. In our future work, we will: (1) examine the molecular pathway explaining how human *MCPH1* copies delay brain development and neural maturation, thus identifying more neoteny-related genes; and (2) apply more sophisticated tests to further understand how the neotenous brain affects cognitive formation in transgenic monkeys.

This study represents the first attempt to utilize a transgenic monkey model to study human brain evolution. It also provides the first molecular genetic evidence showing neotenous changes during brain development caused by human-specific mutations of a single gene. The results highlight the great potential of non-human primate models in studying human evolution and may pave the way for future studies to explore the genetic mechanisms of human-specific traits and elucidate the etiology of human brain disorders such as autism and Alzheimer’s disease.
